# RNA assay identifies a previous misclassification of *BARD1* c.1977A>G variant

**DOI:** 10.1038/s41598-021-02465-y

**Published:** 2021-11-25

**Authors:** Paula Rofes, Marta Pineda, Lídia Feliubadaló, Mireia Menéndez, Rafael de Cid, Carolina Gómez, Eva Montes, Gabriel Capellá, Joan Brunet, Jesús del Valle, Conxi Lázaro

**Affiliations:** 1 Hereditary Cancer Program, Molecular Mechanisms and Experimental Therapy in Oncology (Oncobell) Program, Catalan Institute of Oncology, IDIBELL, Av. Gran Via 199-203, 08908 L’Hospitalet de Llobregat, Spain; 2 Centro de Investigación Biomédica en Red de Cáncer (CIBERONC), 28029 Madrid, Spain; 3 Genomes for Life-GCAT Lab Group, Institut Germans Trias i Pujol (IGTP) (on behalf of the GCAT project), 08916 Badalona, Spain

**Keywords:** Cancer genetics, Gynaecological cancer, Molecular medicine, Breast cancer, Cancer genetics, Gynaecological cancer

## Abstract

Case–control studies have shown an association of *BARD1* with hereditary breast and/or ovarian cancer (HBOC) predisposition. *BARD1* alternatively spliced isoforms are abundant and some are highly expressed in different cancer types. In addition, a number of *BARD1* germline pathogenic variants have been reported among HBOC patients. In previous reports, *BARD1* c.1977A>G variant has been classified as pathogenic since it produces a frameshift transcript lacking exons 2 to 9. In the present study, we sought to validate the mRNA splicing results previously published and to contribute with new evidence to refine the classification of this substitution according to ACMG/AMP guidelines. The presence of the variant was screened in patients and controls. RT-PCR was performed in order to compare the transcriptional profiles of two variant carriers and ten non-carrier controls. In addition, allele-specific expression was assessed. No differences in variant frequency were detected between patients and controls. The RNA assay confirmed the presence of the shorter transcript lacking exons 2–9, but it was detected both in carriers and non-carriers. Furthermore, allelic imbalance was discarded and no significant differences in the proportion of full-length and shorter transcript were detected between carriers and controls. The shorter transcript detected corresponds to *BARD1* isoform η, constituted by exons 1, 10 and 11. Our results support that this transcript is a constitutive splicing product rather than an aberrant transcript caused by *BARD1* c.1977A>G variant, and for this reason this variant should be considered as likely benign following ACMG/AMP guidelines.

## Introduction

BRCA1-associated RING domain 1 (*BARD1*) was first identified in 1996 as a BRCA1-interacting protein^1^. Full-length (FL) *BARD1* transcript comprises 11 exons and encodes a 777 amino acid protein that consists of one N-terminal RING-finger domain, four ankyrin (Ank) repeats and two C-terminal tandem BRCT domains^2,3^. It is through their RING domains that BARD1 and BRCA1 directly interact, mediating double-strand break (DSB) repair as a heterodimer^1,4^. For this reason, *BARD1* has been regarded as a potential breast and/or ovarian cancer predisposing gene and its association with cancer risk has been deeply investigated (reviewed in^5^).

*BARD1* acts as a tumor suppressor, and its expression is necessary to maintain genomic stability and control the cell cycle^6^. However, several oncogenic *BARD1* isoforms have been discovered in different types of cancer with antagonistic effects. The first *BARD1* isoforms were described by Li et al*.* in 2007 associated to human cytotrophoblast invasion and gynecological malignancies^7,8^. Since then, other isoforms have been identified in breast cancer^9^, colorectal cancer^10^, non-small cell lung cancer^11^ and neuroblastoma^12^. These spliced isoforms have been detected both in tumoral and non-tumoral tissues^8–12^, suggesting that *BARD1* alternative splicing is a common feature in human cells^10,13^. Of note, some of these isoforms have been related with a poor prognosis, as they were overexpressed in tumor tissues while FL transcript was underrepresented or absent^8^.

Isoform η is composed of exons 1, 10 and 11. The open reading frame (ORF) is disrupted, but translation can be initiated in an alternative reading frame upstream of the new splice junction^7,8^. Interestingly, the synonymous *BARD1* c.1977A>G has been previously reported as a pathogenic variant that affects splicing^14^, generating an aberrant transcript characterized by the skipping of exons 2 to 9, coinciding with isoform η. The aim of our study was to validate the mRNA splicing results published by Ratajska et al*.* and to provide with new evidence to refine its clinical interpretation according to the American College of Medical Genetics and Genomics and the Association for Molecular Pathology (ACMG/AMP) guidelines.

## Materials and methods

### Patients

A total of 4168 index patients with a personal or family history suggestive of hereditary cancer (HC) referred to the genetic counseling unit at Catalan Institute of Oncology (ICO) hospital were included in the present study. Genetic testing was performed in peripheral blood DNA using our ad hoc NGS custom panel I2HCP, which comprises 122–135 HC-associated genes, depending on the version used^15^. Library preparation methods and bioinformatics pipeline were previously described^15^. The analysis of the panel for diagnostics is phenotype-driven and includes a reduced number of clinically valid and actionable genes associated with a specific tumor type^16^. Informed written consent for both diagnostic and research purposes was obtained from all patients, and the study protocol was approved by the ethics committee of Bellvitge Biomedical Research Institute (IDIBELL; PR278/19). All experiments were performed in accordance with the relevant guidelines and regulations.

### Controls

A set of 194 Spanish cancer-free individuals from the GCAT cohort (GenomesForLife—Cohort Study of the Genomes of Catalonia)^17^ were analyzed with I2HCP panel. In addition, the gnomAD non-Finnish European population, non-cancer data set (Genome Aggregation Database, v2.1.1, http://gnomad.broadinstitute.org/)^18^ was used as a control population.

### Variant nomenclature

Human Genome Variation Society (HGVS) approved guidelines^19^ (http://varnomen.hgvs.org/) were used for *BARD1* variant nomenclature using NM_000465.2 (LRG_297). For variant numbering, nucleotide 1 is the A of the ATG-translation initiation codon.

### In silico analysis

The spliceogenic effect of *BARD1* c.1977A>G variant was evaluated using Alamut Visual software v2.11.0 (Interactive Biosoftware, Rouen, France), which integrates the following prediction methods: Splice Site Prediction by Neural Network^20^ (NNSPLICE), SpliceSiteFinder-like^21^ (SSF), MaxEntScan^22^ (MES) and GeneSplicer^23^ (GS). All algorithms were used with the default settings. In addition, SpliceAI software was also used^24^.

### RNA analysis

**Figure 1:**
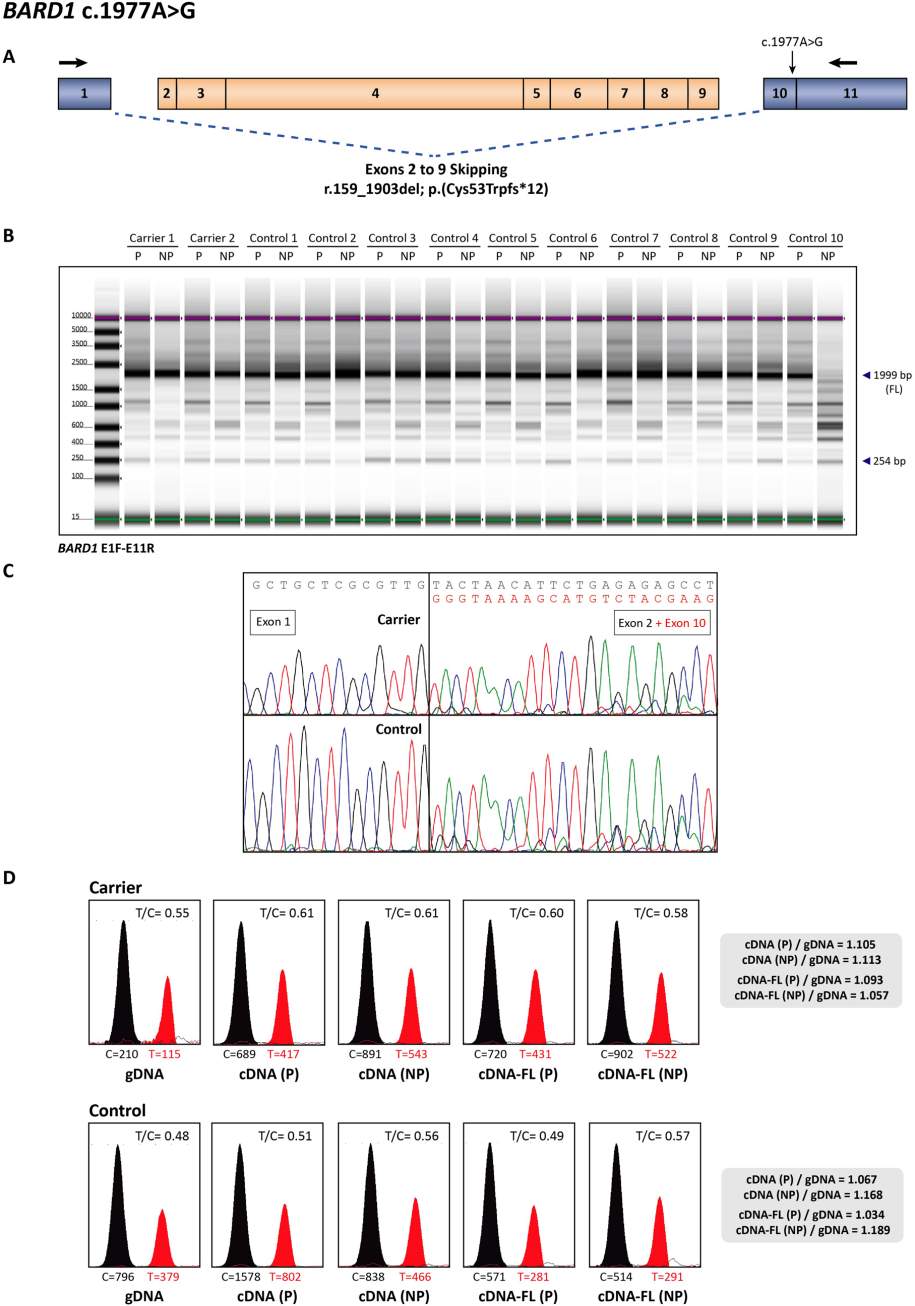
mRNA splicing assay of *BARD1* c.1977A>G variant. (**A**) Schematic representation of a shorter splicing transcript detected by the mRNA assay. Skipped exons are colored in orange, and blue discontinuous lines represent the skipping pattern. Black arrows represent the location of forward and reverse primers. (**B**) TapeStation assay of two patients who harbored *BARD1* c.1977A>G variant (carriers 1 and 2) and ten non-carrier controls. cDNA amplification of exons 1–11 manifested several bands, the upper one corresponding to the full-length (FL) transcript (1999 bp) and the lower to the transcript lacking exons 2 to 9 (254 bp). P: sample treated with puromycin prior to RNA extraction; NP: sample not treated with puromycin; FL: full-length; bp: base pairs; F: forward; R: reverse. (**C**) Sanger sequencing results of one variant carrier and one non-carrier control. In the electropherogram, reference sequences are displayed in gray, whereas the alternative sequences are represented in red. (**D**) Single-nucleotide primer extension assay (SNuPE) results in one patient and one control sample. Allele-specific expression (ASE) was evaluated using exon 6 *BARD1* c.1519G>A polymorphism. C and T values displayed under the peaks correspond to peak heights; T/C ratios represent the proportion of variant/wild-type allele in each sample; ASE values are displayed in the gray square, calculated as the proportion of variant/wild-type allele in cDNA by the proportion of variant/wild-type allele in gDNA. P: sample treated with puromycin prior to RNA extraction; NP: sample not treated with puromycin; cDNA-FL: cDNA analysis on the full-length excised band.

Lymphocytes were isolated by Ficoll gradient centrifugation of peripheral blood samples from two patients who harbored *BARD1* c.1977A>G variant and ten non-carrier controls. Cells were cultured in PB-Max medium for 5–7 days in two subcultures, one of which was treated with puromycin 4–6 h before RNA extraction in order to prevent the potential degradation of unstable transcripts by nonsense-mediated decay (NMD). Total RNA was isolated using TRIzol reagent and reverse transcribed with iScript cDNA Synthesis kit (Bio-Rad Laboratories, Hercules, CA, USA). cDNA amplification was performed with the following primers: exon 1 forward primer 5′-CCATGGAACCGGATGGTC-3′ and exon 11 reverse primer 5′-AGGTTGTCCTTTGGATGGTG-3′ (Fig. 1A). Transcriptional profiles from variant carriers were compared with those derived from ten control lymphocyte cultures by agarose gel analysis, TapeStation Assay analysis (Agilent 4200 TapeStation System, Agilent Technologies, Santa Clara, CA, USA) (Fig. 1B) and Sanger sequencing (Fig. 1C)^25^.

### Quantification of full-length transcripts produced by the variant allele

In order to evaluate the extent of the splicing effect, bands corresponding to the FL transcript were excised from agarose gels and single-nucleotide primer extension (SNuPE) was performed to assess allele-specific expression (ASE) using *BARD1* c.1519G>A polymorphism as tag-SNP (Fig. 1D). Briefly, gene-specific primers were used to amplify the region enclosing the targeted variant, both in gDNA and cDNA of one carrier and one control. gDNA amplification was performed with 5′-TCCATTGCTCTTTCTTATCACTTC-3′ forward primer and 5′-TCTGCTTTATCACACACCTTGA-3′ reverse primer, whereas cDNA amplification was performed with 5′-CCATGGAACCGGATGGTC-3′ forward primer and 5′-AGGTTGTCCTTTGGATGGTG-3′ reverse primer. PCR products were purified using GFX PCR DNA and Gel Band Purification kit (GE Healthcare, Chicago, IL, USA), and then used in the SNaPshot quantitative primer extension assay according to the manufacturer’s protocol (SNaPshot Multiplex kit, ThermoFisher Scientific, Waltham, MA, USA). The primer used for the SNuPE assay was: 5′-AACAGCTTGACTATATCCA-3′. Following cleanup with SAP (shrimp alkaline phosphatase), products of the primer extension reaction were separated on an ABI 3130xl Genetic Analyzer (Applied Biosystems, Foster City, CA, USA). Data were analyzed using GeneMapper v4.0 (Applied Biosystems) and ASE was calculated by dividing the proportion of variant/wild-type allele in cDNA by the proportion of variant/wild-type allele in gDNA^25^. All experiments were performed with three replicates.

### Variant classification

The ACMG/AMP guidelines were used for variant interpretation^26^.

## Results

### Prevalence of *BARD1* c.1977A>G variant in HC and control cohorts

Multi-gene panel testing was performed in a total of 4168 HC patients, and *BARD1* carrier status was retrieved from all individuals in a research context for this study. Twenty patients harboring *BARD1* c.1977A>G variant were identified, representing a carrier frequency of 0.5%. Of these, twelve patients were recruited with a clinical suspicion of hereditary breast and/or ovarian cancer, seven with a clinical suspicion of hereditary colorectal cancer and one with a clinical suspicion of melanoma (Table 1). In addition to *BARD1* c.1977A>G, only one patient also harbored *BRCA1* c.68_69del; p.(Glu23Valfs*17) pathogenic variant. Among 194 Spanish control samples, one carrier of the variant was identified (carrier frequency = 0.5%). Furthermore, 354 individuals from gnomAD non-cancer database harbored *BARD1* c.1977A>G variant (carrier frequency = 0.6%), one of them in homozygosis. Therefore, the frequency of the variant in the HC cohort was comparable to that of the Spanish control cohort and gnomAD non-cancer cohort (Table 1).

**Table 1:** *BARD1* c.1977A>G carriers identified in a hereditary cancer cohort (classified by their clinical suspicion) and two control populations.

	Number of carriers	Total of individuals	Frequency (%)
**Clinical suspicion**			
Hereditary colorectal cancer	7	966	0.7
Hereditary breast cancer	6	1688	0.4
Hereditary ovarian cancer	5	551	0.9
Hereditary breast and ovarian cancer	1	435	0.2
Melanoma	1	119	0.8
Total hereditary cancer cohort	20	4168	0.5
**Control cohorts**			
GCAT Spanish population cohort	1	194	0.5
gnomAD non-Finnish European, non-cancer dataset	354	59,056	0.6

### mRNA splicing assay results

Total RNA was isolated from cultured lymphocytes of two patients who harbored *BARD1* c.1977A>G variant and ten non-carrier controls, and their transcriptional profiles were compared by agarose and capillary electrophoresis analyses (Fig. 1B,C)^25^. As previously reported by Ratajska et al*.*^14^, a transcript lacking exons 2 to 9 was detected (r.159_1903del), but it was observed in both variant carriers and controls. This transcript presumably leads to a truncated protein (p.(Cys53Trpfs*12)) (Fig. 1A). However, only minor traces of this shorter transcript were detected in comparison to the FL (Fig. 1C). Contrarily to the study published by Ratajska et al*.*, the presence of this shorter transcript was confirmed in all controls (Fig. 1B).

In order to elucidate whether the splicing effect was total or partial, allele-specific expression (ASE) was evaluated. To this aim we analyzed the expression of one patient and one control that harbored the *BARD1* c.1519G>A polymorphism, located in the skipped region (exon 6). The proportion of variant and wild-type allele in cDNA did not differ from that detected in gDNA, and no significant differences were detected between patient and control samples. Furthermore, biallelic expression was detected after excision of the FL band (Fig. 1D). Therefore, allele-specific imbalance was discarded.

Although other transcripts were also detected, their size distribution and expression levels were equivalent between HC patients and controls (Fig. 1B,C). Therefore, they were disregarded as aberrant transcripts associated with c.1977A>G variant.

### Variant interpretation following ACMG/AMP guidelines

The guidelines published by ACMG/AMP^26^ are based on scoring 28 different criteria of pathogenicity (P) or benignity (B) to interpret sequence variants. Each criterion is in turn ascribed with different strength levels: very strong (VS), strong (S), moderate (M) or supporting (P).

PS3 criterion can be used when well-established in vitro or in vivo functional studies are supportive of a damaging effect on the gene or gene product. In a previous publication by our group^25^, we devised a proposal on how to weight this evidence according to the results observed in in vitro RNA assays. The predominance of the FL transcript from the variant allele detected in HC patients, the low proportion of the shorter transcript observed, and the same splicing pattern detected in ten controls, suggested the alternative transcript r.159_1903del as a constitutive splicing product. *BARD1* c.1977A>G variant is a synonymous substitution without impact at the protein level, p.Arg659=. Our results further discard a potential splicing alteration, r.1977a>g, since no damaging effect on the gene or gene product was detected and allele-specific silencing was dismissed. Therefore, the variant was weighted with BS3 criterion.

PP3 criterion is used when computational evidence supports a deleterious effect, and when no impact on gene or gene product is suggested BP4 is used instead. In this case, the variant was evaluated using five different in silico algorithms, and no deleterious effect was computationally predicted (Supplemental Table 1). Therefore, *BARD1* c.1977A>G was weighted with BP4 criterion.

Regarding population data, *BARD1* c.1977A>G variant is present in approximately 1 in 170 individuals with European ancestry, being more common than most cancer-predisposing variants. This could explain the reason why most ClinVar submissions classify this variant as (likely) benign, disregarding the functional assay published by Ratajska et al*.*^14^. BS1 criterion can be applied when population data is not consistent with disease prevalence. Despite the association of *BARD1* with breast cancer risk^27^, gnomAD data suggest a greater tolerance for loss-of function variants in *BARD1* (observed/expected ratio = 1.03 (90% Confidence Interval = 0.79–1.36)). Consequently, due to difficulty in establishing a threshold for BS1 criterion for this gene, it was disregarded for *BARD1* c.1977A>G variant.

Following ACMG/AMP guidelines, the combination of all the evidences collected (BS3 + BP4) supports the classification of *BARD1* c.1977A>G substitution as a likely benign variant.

## Discussion

*BARD1* was postulated as a hereditary breast and ovarian cancer (HBOC) predisposing gene shortly after it was first described, due to its relationship with *BRCA1* in terms of shared structural homology and functional association for the development of their tumor-suppressor roles^1^. However, its role in cancer susceptibility remains inconclusive, and the results obtained in several case–control studies have been controversial so far^5^. We have recently published a case–control study investigating the role of *BARD1* in cancer predisposition in a Spanish HBOC cohort of 4015 individuals. The results supported a significant association of *BARD1* PVs with hereditary breast cancer (OR = 4.18; CI = 2.10–7.70; p = 5.45 × 10^–5^), particularly among triple-negative tumors (OR = 5.40; CI = 1.77–18.15; p = 0.001)^28^. However, it should be noted that only truncating and canonical splice site variants were considered for the risk calculations, whose prevalence in breast and/or ovarian cancer cases is very limited. As a result of the lack of *bona fide* protein functional studies, missense, synonymous and intronic variants have not yet been contemplated in most association studies. These three types of variants can be potentially spliceogenic, as the introduction of a DNA sequence alteration can lead to the disruption of canonical splice sites, as well as the activation or creation of other cryptic splice donors or acceptors^29^. As a consequence of an aberrant splicing pattern, transcripts carrying loss-of-function (LoF) alterations associated with the disease could be generated.

This article is focused on the characterization of *BARD1* c.1977A>G variant. A previous study published by Ratajska et al*.*^14^ identified a shorter transcript characterized by the skipping of exons 2 to 9 (r.159_1903del; p.(Cys53Trpfs*12)) in an ovarian cancer patient harboring this variant. The presence of this transcript was discarded in one non-carrier control, thus it was attributed to the variant studied. Nevertheless, the analysis of ten non-carrier controls of our cohort has evidenced the presence of this shorter transcript in all of them, and therefore it cannot be associated to *BARD1* c.1977A>G variant. Due to the inconsistencies in variant classification across different laboratories, the ENIGMA Consortium Splicing Working group defined some reporting guidelines to ensure the standardization of splicing assay protocols, as well as the interpretation of the data obtained^30^. Accordingly, mRNA assays from patient samples should be compared to at least ten reference controls, in order to facilitate the identification of naturally occurring isoforms. Besides, our study has evidenced the utility of highly sensitive electrophoretic analyses, since the limited resolution of conventional agarose gels could hinder the detection of less abundant alternative transcripts. Sanger sequencing should always be performed in order to assess the presence and to accurately characterize all transcripts, not only in variant carriers, but also in controls^25^.

We have also performed an ASE assay using *BARD1* c.1519G>A polymorphism, which is located in the skipped region. Both alleles were equally expressed in total cDNA and in the FL band in carriers and controls, which points out to a physiologic alternative splicing and rules out an allelic imbalance. Furthermore, the shorter transcript does not appear to be enriched in patients, as no differences were detected between carriers and controls. In this shorter transcript, translation could be initiated in an alternative ORF^8^ that would result in an in frame deletion. Consequently, NMD would not be triggered, which is supported by our results as no differences were detected between samples treated with puromycin and untreated samples.

Another remarkable feature is that there do not seem to be differences in the prevalence of *BARD1* c.1977A>G variant between patients and the general population. On one side, we identified *BARD1* c.1977A>G substitution as the most prevalent *BARD1* variant in our HC cohort, with 20 carriers among 4168 individuals (carrier frequency = 0.5%). Interestingly, its presence was not restricted to HBOC patients, as one would expect considering the association of *BARD1* with breast and/or ovarian tumors. Besides, one out of 194 geographically-matched controls (GMCs) also harbored the same variant (carrier frequency = 0.5%). The comparison with a large-scale dataset evidenced that there were no significant differences between patients and controls (carrier frequency in gnomAD non-cancer cohort = 0.6%), indicating that this variant does not appear to be particularly associated with cancer. Unfortunately, the lack of association between a variant and disease does not constitute a criterion in the current ACMG/AMP classification guidelines.

Although nearly 95% of mammalian genes undergo alternative splicing^31^, it is essential to differentiate between naturally occurring isoforms and aberrant splicing events. *BARD1* splice variants are abundant^13^ and several isoforms (including α, β, κ, γ, δ, φ, ε, η, π and ω) with antagonistic effects have been found to be highly expressed in various cancers^7–12^. Since isoform η also lacks exons 2 to 9 and taking into account all the evidence collected in this study, we believe that the skipped transcript detected corresponds to the constitutive splicing product named isoform η, equally expressed in variant carriers and controls. According to ACMG/AMP guidelines, our results reinforce the reinterpretation of this variant as (likely) benign, in consistency with most of its current ClinVar classifications.

In conclusion, our data does not support that *BARD1* c.1977A>G variant promotes any aberrant splicing that could be associated with disease, as we have detected the same constitutive transcript in variant carriers and non-carrier controls. Furthermore, the frequency of this variant is similar in patients and in the general population, ruling out an increased risk of cancer associated to this variant.

## Supplementary Information

Supplementary Table 1.

## Data Availability

All data generated or analyzed during this study are included in this published article and its supplementary information files.
